# Extended working hours, income, and multidimensional wellbeing among physicians in China: a cross-sectional mediation analysis

**DOI:** 10.3389/fpubh.2026.1897935

**Published:** 2026-08-26

**Authors:** Huixian Zheng, Mingyue Li, Haoqing Tang, Yuxun Zhou, Xiaoyun Liu

**Affiliations:** 1 Department of Health Policy and Management, School of Public Health, Peking University, Beijing, China; 2 China Center for Health Development Studies, Peking University, Beijing, China; 3 Beijing Institute for Health Development, Peking University, Beijing, China; 4 National Health Commission Key Laboratory of Health System Reform and Governance (Peking University), Beijing, China; 5 WHO Collaborating Centre for Universal Health Coverage, Beijing, China

**Keywords:** income, performance-based compensation system, physicians, productivity, wellbeing

## Abstract

**Background:**

Physician wellbeing is central to healthcare workforce sustainability, particularly in settings characterized by high work demands and extended working hours. In China's healthcare system, physician income remains an important occupational factor and may be linked to workload and service volume, adding complexity to the associations between working hours, income, and wellbeing. This study examined these associations and the potential mediating role of income among physicians in China.

**Methods:**

A cross-sectional survey was conducted among 1,324 physicians from four province-level regions in China. Physician wellbeing was measured across physiological, psychological, and work-related domains. Generalized Structural Equation Modeling (GSEM) with 5,000 bootstrap replications was employed to estimate the direct associations between weekly working hours and multidimensional wellbeing and the income-mediated indirect associations.

**Results:**

The mean score for physiological wellbeing (EQ-VAS) was 70.2 ± 16.7. Overall, 42.3% of physicians reported poor psychological wellbeing, defined as anxiety or depression, and 72.4% did not meet the criteria for good work-related wellbeing. Longer weekly working hours were inversely associated with physiological wellbeing (*B* = −5.057, *p* < 0.001), psychological wellbeing (OR = 0.685, *p* < 0.001), and work-related wellbeing (OR = 0.481, *p* < 0.001). Longer weekly working hours were also positively associated with monthly income (*B* = 0.049, *p* < 0.01). Income-mediated indirect associations were positive for physiological wellbeing (indirect association = 0.180; 95% bootstrap CI: 0.044, 0.368) and psychological wellbeing (indirect OR = 1.020; 95% bootstrap CI: 1.005, 1.041), but not for work-related wellbeing (indirect OR = 1.011;95% bootstrap CI: 0.998, 1.032).

**Conclusion:**

Among physicians surveyed in China, longer weekly working hours were associated with poorer multidimensional wellbeing. Monthly income played a modest statistical mediating role in the associations with physiological and psychological wellbeing, while the corresponding indirect association was not evident for work-related wellbeing under the primary strict definition. Further longitudinal and policy-evaluation studies are warranted to inform work-hour management and compensation reforms.

## Introduction

Physicians constitute a central pillar of any healthcare system, serving as direct providers of essential health services and playing a vital role in safeguarding patient safety and public health (1, 2). Consequently, healthcare workforce sustainability relies on maintaining both physician wellbeing and adequate service capacity, with physicians' work input and service delivery commonly reflected in working hours and service volume (3). Extensive research has shown that these two dimensions are closely interconnected: compromised wellbeing, particularly burnout, has been linked to increased error rates, reduced service quality, and operational inefficiency (4). For instance, studies indicate that elevated emotional exhaustion increases the probability of reporting major medical errors by 6% to 10% (5). Therefore, while sufficient work input is necessary to maintain service capacity, it must be balanced against the physiological and psychological limits of the medical workforce to sustain quality of care.

Compensation and financial incentives are central considerations in healthcare workforce management and are closely intertwined with physicians' work input, productivity and wellbeing (6). Similar to global practices (7), China has implemented performance-based compensation arrangements across public medical and public health institutions, including public hospitals, primary healthcare centers (PHCs), and Centers for Disease Control and Prevention (CDCs) (8, 9). By linking a portion of remuneration to workload- and service-quality-related indicators, these arrangements are intended to strengthen the connection between work input and pay and to provide performance-related incentives for healthcare workers (8).

However, the implementation of these arrangements is shaped by the financial circumstances of medical institutions. Facing fiscal pressures to cover operational costs and deficits (10), many institutions increasingly prioritize efficiency and productivity (11). This is particularly relevant in public hospitals, where regulated basic salaries remain relatively modest and performance-related payments constitute an important component of physicians' income (8). Against this institutional background, greater work input, reflected in longer working hours and heavier workloads, may be associated with higher income (12).

These economic incentives operate within a context where workload pressures are further compounded by structural factors. Although the overall physician density in China is comparable to the median of the Organization for Economic Co-operation and Development (OECD) countries (13), the distribution of qualified personnel remains highly uneven (14). Furthermore, rapid population aging and a fragmented tiered health-care delivery system often lead patients to bypass primary care facilities, causing significant patient surges in higher-level hospitals (15). This creates a challenging environment where hospital physicians grapple with excessive clinical volume driven by patient demand, while those in primary care and disease control settings often face resource scarcity and insufficient support (16, 17).

Together, these compensation arrangements and structural pressures create a context in which extended working hours are common among physicians in China. Recent national data indicate that nearly one third of Chinese physicians work more than 60 h per week (18). This situation highlights a potential tension between the higher income that may accompany greater work input and concerns regarding physician wellbeing. However, the role of monthly income in the relationship between working hours and physician wellbeing remains insufficiently understood. Examining this relationship is important for informing healthcare workforce management and remuneration policy in China.

Recent healthcare management research has examined healthcare sustainability and workforce outcomes from broader organizational and managerial perspectives, including quality-management practices, innovative culture, organizational politics, employee resilience, and person-job fit (19, 20). Collectively, this literature underscores the importance of organizational and workplace conditions for healthcare workforce outcomes. Within this broader field, however, work input and financial rewards have often been examined separately. Previous studies have linked long working hours with burnout, cardiovascular risks, and lower subjective wellbeing (21), whereas higher income has been associated with job satisfaction and retention (22). Few studies have integrated these lines of research to examine whether monthly income statistically mediates part of the association between extended working hours and physician wellbeing.

Physician wellbeing is increasingly recognized as multidimensional, yet its definition and measurement remain inconsistent (23). Previous research has often emphasized negative outcomes, particularly burnout, while giving less attention to physiological and broader psychosocial aspects of wellbeing (23–25). Guided by the World Health Organization's broad conception of health (26), the present study assesses physician wellbeing across three complementary domains: physiological wellbeing, reflected in perceived overall health; psychological wellbeing, reflected in symptoms of anxiety and depression; and work-related wellbeing, reflected in burnout, job satisfaction, and work-life balance. This domain-specific approach allows us to examine whether the associations with working hours and income vary across different aspects of physician wellbeing.

The Job Demands-Resources (JD-R) theory (27) provides an explanatory framework for understanding the association between working hours and physician wellbeing. In this framework, extended working hours can be conceptualized as a quantitative job demand requiring sustained physical and psychological effort. Consistent with Conservation of Resources theory (28), prolonged exposure to such demands may deplete physicians' physical and psychological resources and reduce opportunities for recovery. Therefore, longer weekly working hours are expected to be negatively associated with each of the three wellbeing domains, providing the theoretical basis for H1.

The effort-reward perspective further emphasizes the balance between work effort and the monetary and status-related rewards received in return (29). In settings where income includes performance-related components, longer working hours may be associated with higher monthly income (30). Previous research has linked higher pay or income with greater job satisfaction, better self-rated health, and more favorable effort-reward conditions (31–34). Taken together, these theoretical and empirical findings provide a rationale for examining whether monthly income statistically mediates part of the associations between weekly working hours and physician wellbeing, thereby providing the basis for H2a and H2b.

Against this background, this study examined the associations among weekly working hours, income, and physiological, psychological, and work-related wellbeing using data from physicians in four province-level regions in China. Specifically, we assessed whether monthly income statistically mediated part of the associations between weekly working hours and each wellbeing domain.

The study addressed the following research questions:

RQ1: How are weekly working hours associated with the three physician wellbeing domains?

RQ2: How are weekly working hours associated with monthly income?

RQ3: Does monthly income statistically mediate part of the associations between weekly working hours and the three wellbeing domains?

**Figure 1:**
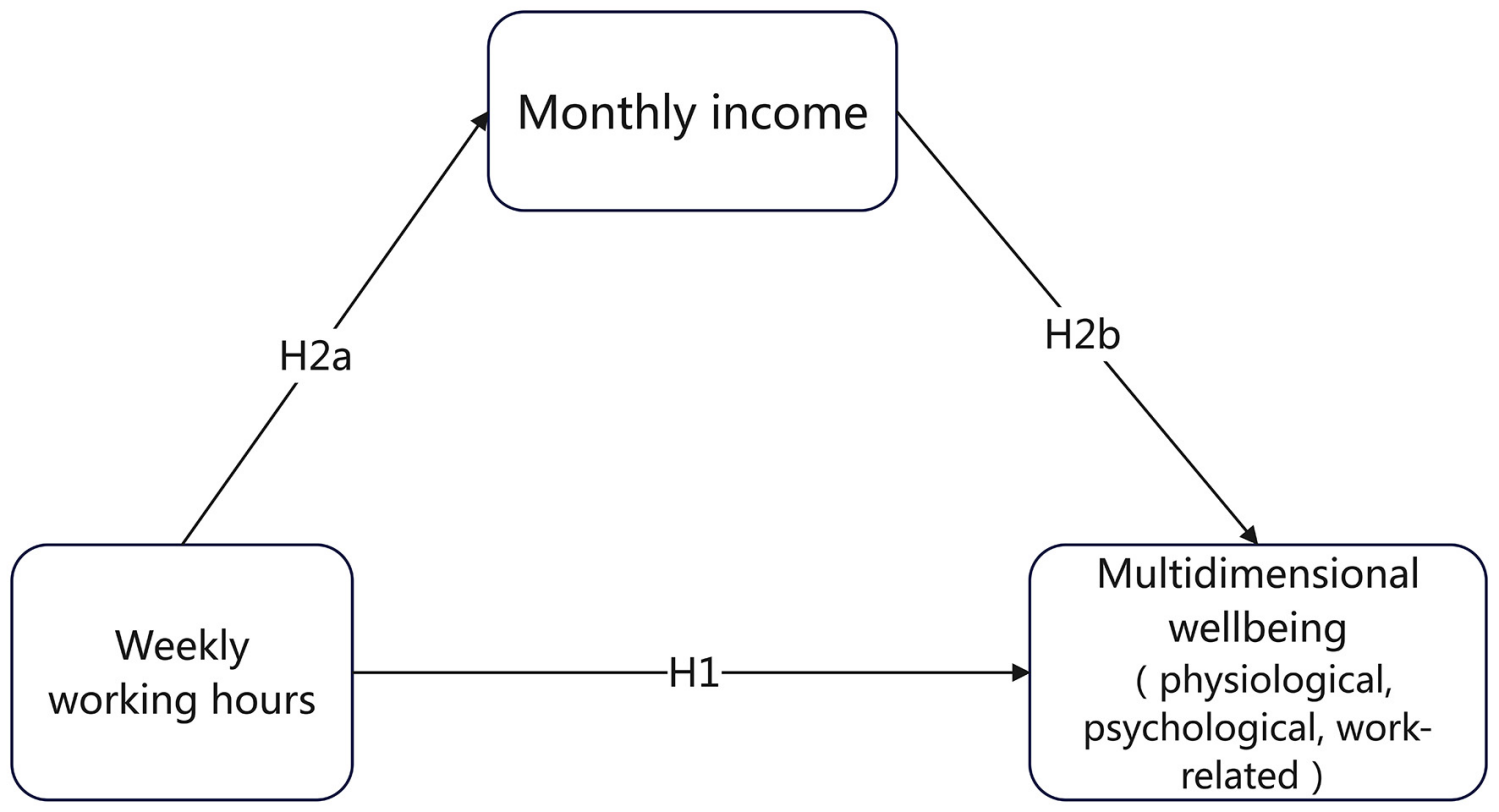
Conceptual framework of the study.

This study contributes to the literature in two respects. First, it connects research on work demands and financial rewards by examining an income-related indirect statistical pathway within a single analytical framework. Second, it adopts a domain-specific assessment of physician wellbeing rather than relying on burnout or another single outcome, allowing the associations with working hours and income to be compared across physiological, psychological, and work-related domains. The proposed conceptual model is presented in Figure 1.

H1: Longer weekly working hours are negatively associated with each of the three wellbeing domains.

H2a: Longer weekly working hours are positively associated with monthly income.

H2b: Monthly income statistically mediates part of the associations between weekly working hours and each wellbeing domain.

## Methods

### Study design, sampling, and participants

A cross-sectional survey was conducted from July to October 2024. To capture variation in socioeconomic development and healthcare resource distribution, we selected four province-level regions in China: Shanghai and Tianjin, two eastern municipalities characterized by relatively high economic development and dense healthcare resources; Henan Province, including Xinxiang and Luoyang, representing a populous central province with substantial healthcare service demands; and Shaanxi Province, represented by Xi**'**an, a major regional medical center in western China. This geographical layout was intended to include diverse healthcare contexts across regions with different levels of resource concentration and service demand.

A multi-stage non-proportional stratified sampling strategy was employed to include physicians from different tiers of the healthcare system. In the first stage, survey sites were purposively selected in each region according to facility type and local coordination capacity. The planned sites included one tertiary general hospital, one secondary general hospital, one CDC, and three to four primary healthcare facilities in each region. In the second stage, physician-level quota sampling was applied to achieve adequate sample sizes across facility types, particularly for smaller strata such as CDCs. Quotas were set based on statistical power and operational feasibility considerations. The target quotas per region were approximately 150 physicians from tertiary hospitals, 50 from secondary hospitals, 50 from CDCs, and a cumulative total of 50–60 from primary healthcare centers, with approximately 15–20 physicians drawn from each site.

The target population comprised licensed physicians, including licensed assistant physicians, who were on duty on the day of the survey. In accordance with the *Physicians Law of China*, physicians were defined to include practitioners in clinical medicine, Traditional Chinese Medicine (TCM), stomatology, and public health (35). Physicians on long-term leave, such as sick leave, maternity leave, or advanced training, were excluded. Based on the sampling design, the planned total sample size was approximately 1,000 physicians across the selected regions.

### Data collection and quality control

Data were collected on site using an anonymous electronic questionnaire administered through WenjuanXing, a widely used online survey platform in China. The survey was implemented in two modes: group-administered completion and individually self-administered completion. In the group-administered mode, local coordinators gathered physicians in a central location where investigators introduced the study protocol, provided instructions, answered questions, and invited participants to scan a QR code to complete the questionnaire. In the individually self-administered mode, investigators coordinated with institutional and departmental administrators to provide physicians with a study-specific QR code, allowing them to complete the questionnaire during their work breaks. In both modes, at least two investigators were present on site to oversee implementation and monitor questionnaire submissions through the WenjuanXing platform in real time. Electronic informed consent was embedded on the first page of the questionnaire, and participants could proceed to the survey only after confirming consent. The study protocol was approved by the Peking University Health Science Center's Institutional Review Board (IRB00001052-24072).

Quality control procedures were implemented during survey administration and subsequent data cleaning. During fieldwork, investigators observed the questionnaire completion process and monitored incoming submissions through the WenjuanXing platform in real time. The platform was configured to allow only one submission per device or WeChat account, to reduce the likelihood of duplicate submissions. After data collection, all submitted questionnaires were reviewed according to predefined exclusion criteria. Questionnaires completed in less than 10 min were considered indicative of careless responding and were removed. Submissions were also excluded if they lacked key research information, particularly responses to the burnout scale, or if the overall item completion rate was below 80%. In addition, responses with obvious logical inconsistencies, such as implausible combinations of age and years of service, or patterned response styles such as straight-lining, were excluded to improve the reliability of the final analytic dataset. A total of 1,463 questionnaire submissions were recorded on the WenjuanXing platform. After applying the quality control criteria, 1,324 submissions were retained in the final analytic sample, representing 90.5% of all submitted questionnaires.

### Measurement of variables

#### Multidimensional physician wellbeing

Physician wellbeing was assessed across physiological, psychological, and work-related dimensions. Physiological wellbeing was measured using the EuroQol Visual Analogue Scale (EQ-VAS) (36), on which respondents rated their overall health status on a continuous scale ranging from 0 for the worst imaginable health to 100 for the best imaginable health. Psychological wellbeing was assessed using the anxiety/depression item from the EQ-5D-3L (37). While distinct from multi-dimensional clinical diagnostic tools, this validated single-item measure was selected to efficiently capture respondents' immediate psychological state while minimizing survey fatigue. Specifically, participants were asked: “Did you feel anxious or depressed today?” For analysis, respondents who reported no anxiety or depression were classified as having good psychological wellbeing (coded as 1). Work-related wellbeing was operationalized as a conservative composite indicator derived from job satisfaction, work-life balance, and the absence of burnout. Job satisfaction and work-life balance were each evaluated using a single-item five-point Likert scale. For job satisfaction, responses of “Satisfied” or “Very Satisfied” were classified as positive; for work–life balance, responses of “Agree” or “Strongly Agree” were classified as positive. Burnout was measured using the Maslach Burnout Inventory-Human Services Survey (MBI-HSS) (38), defined as an emotional exhaustion score of 27 or higher or a depersonalization score of 10 or higher. The three subscales showed good internal consistency in this sample (Cronbach's α: emotional exhaustion = 0.906, depersonalization = 0.769, and personal accomplishment = 0.902). A composite score ranging from 0 to 3 was constructed by summing these three positive states. Under the primary strict definition, good work-related wellbeing required meeting all three criteria simultaneously (39).

#### Measurement of working hours and income

Weekly working hours served as the primary predictor, and monthly income was examined as the mediator. To reduce respondent burden and sensitivity, data for these variables were collected using categorical ranges rather than exact values. Participants selected the corresponding intervals to report their average weekly working hours over the past month and their average monthly work-related after-tax income over the past year, which included basic salary, performance bonuses, and allowances, but excluded social insurance and other statutory deductions. For the purpose of statistical analysis, these categorical variables were converted into continuous variables by assigning the midpoint value of each selected range as the representative estimate for that observation (40).

#### Statistical analysis

Statistical analyses were performed using Stata 18.0. Descriptive statistics were used to summarize the sample characteristics, with continuous variables expressed as mean ± standard deviation (SD) and categorical variables as frequencies and percentages. Differences across institutional types were assessed using chi-square tests for categorical variables and Kruskal-Wallis tests for continuous variables. Spearman's rank correlation analysis was performed to examine bivariate associations among weekly working hours, monthly income, and multidimensional wellbeing.

To improve the interpretability of the regression coefficients, weekly working hours were rescaled into 10-h units; thus, the corresponding coefficient represents the estimated change associated with a 10-h increase in weekly working hours. This linear transformation does not alter the correlation coefficients in the Spearman correlation analysis. Monthly income was log-transformed in the regression and mediation analyses. The primary multivariable models adjusted for gender, age group, province, and institution type. Linear regression was used for physiological wellbeing, while logistic regression was used for psychological and work-related wellbeing. Robust standard errors were applied in these primary models.

Mediation analyses were conducted using Generalized Structural Equation Modeling (GSEM) with 5,000 bootstrap replications (41), and were interpreted as a statistical decomposition of direct and income-mediated indirect associations. For each wellbeing outcome, the overall association with weekly working hours was first estimated using a reduced-form model excluding monthly income as the mediator. Direct and income-mediated indirect associations were then estimated using GSEM. Estimates were reported as unstandardized coefficients for physiological wellbeing and as odds ratios (ORs) for psychological and work-related wellbeing; indirect ORs were calculated by exponentiating the corresponding logit-scale product terms. Confidence intervals (CIs) for the reduced-form overall associations were based on robust standard errors, whereas CIs for the GSEM-based direct and income-mediated indirect associations were percentile-based 95% bootstrap CIs. For GSEM-based direct and income-mediated indirect associations, statistical significance was indicated when the 95% CI excluded the null value, which was 0 for unstandardized coefficients and 1 for ORs.

Several supplementary analyses evaluated the robustness of the findings. First, a relaxed definition of work-related wellbeing was examined, requiring individuals to meet at least two of the three favorable component criteria. Second, component-specific analyses treated job satisfaction, work-life balance, and absence of burnout as separate binary outcomes. Third, expanded-adjustment models additionally included facility category, education, and professional title. In separate alternative specifications, broad professional field group replaced facility category, and years of practice replaced age group. Multicollinearity in the primary adjustment model and the three expanded-adjustment models was assessed using variance inflation factors (VIFs). Finally, to account for potential within-cluster correlation, the primary regression models were re-estimated with cluster-robust standard errors using a cluster identifier based on the organizational structure of participant recruitment. The identifier comprised 25 clusters, while province indicators were retained in the models. For conventional regression-based estimates, statistical significance was defined as a two-sided *p*-value < 0.05.

## Results

Table 1 describes the summary statistics of the entire study sample (*N* = 1,324) and compares characteristics by institutional type. The sample was predominantly female (59.5%), with physicians aged 35–49 years constituting the largest group (50.2%). Geographically, participants were recruited from Henan (34.5%), Shaanxi (22.4%), Shanghai (22.0%), and Tianjin (21.1%). Regarding institutional distribution, 57.1% were employed in hospitals, 22.7% in primary healthcare centers, and 20.2% in CDCs. In terms of professional background, over half of the physicians (54.8%) held a postgraduate degree, and 45.3% held an intermediate professional title. The mean weekly working hours were 49.4 h, and the mean monthly income was CNY 8,245.1. Differences across the three institutional types were observed in gender, age, education level, professional title, weekly working hours, and monthly income (*p* < 0.05).

**Table 1:** Summary statistics of the sample (*n*, %).

Variables	PHCs	Hospitals	CDCs	Total	*χ^2^/H*	*p*
	***N*** = **301**	***N*** = **756**	***N*** = **267**	***N*** = **1,324**		
Gender						
Female	224 (74.4%)	385 (50.9%)	179 (67.0%)	788 (59.5%)	57.17	< 0.001
Male	77 (25.6%)	371 (49.1%)	88 (33.0%)	536 (40.5%)		
Age						
< 35 years	72 (23.9%)	288 (38.1%)	131 (49.1%)	491 (37.1%)	63.52	< 0.001
35–49 years	171 (56.8%)	401 (53.0%)	92 (34.5%)	664 (50.2%)		
≥50 years	58 (19.3%)	67 (8.9%)	44 (16.5%)	169 (12.8%)		
Educational level						
Associate degree or below	34 (11.3%)	5 (0.7%)	11 (4.1%)	50 (3.8%)	290.06	< 0.001
Bachelor's degree	224 (74.4%)	226 (29.9%)	98 (36.7%)	548 (41.4%)		
Postgraduate degree	43 (14.3%)	525 (69.4%)	158 (59.2%)	726 (54.8%)		
Province						
Shaanxi	79 (26.2%)	162 (21.4%)	55 (20.6%)	296 (22.4%)	10.46	0.107
Henan	93 (30.9%)	272 (36.0%)	92 (34.5%)	457 (34.5%)		
Shanghai	77 (25.6%)	160 (21.2%)	54 (20.2%)	291 (22.0%)		
Tianjin	52 (17.3%)	162 (21.4%)	66 (24.7%)	280 (21.1%)		
Professional title						
Junior or below	90 (29.9%)	205 (27.1%)	74 (27.7%)	369 (27.9%)	14.47	0.006
Intermediate	149 (49.5%)	349 (46.2%)	102 (38.2%)	600 (45.3%)		
Senior	62 (20.6%)	202 (26.7%)	91 (34.1%)	355 (26.8%)		
Weekly working hours [mean (SD), hours/week]	43.4 (6.4)	53.8 (9.6)	43.6 (6.4)	49.4 (9.8)	372.51	< 0.001
Monthly income [mean (SD), ¥]	6,302.3 (3,598.8)	9,363.1 (4,786.3)	7,269.7 (3,315.3)	8,245.1 (4,470.5)	128.59	< 0.001
Wellbeing indicators						
Physiological wellbeing [mean (SD)]	69.3 (17.0)	69.8 (16.9)	72.2 (15.8)	70.2 (16.7)	5.61	0.061
Psychological wellbeing						
Poor	118 (39.2%)	327 (43.3%)	115 (43.1%)	560 (42.3%)	1.53	0.465
Good	183 (60.8%)	429 (56.7%)	152 (56.9%)	764 (57.7%)		
Work-related wellbeing						
Poor	199 (66.1%)	559 (73.9%)	201 (75.3%)	959 (72.4%)	7.97	0.019
Good	102 (33.9%)	197 (26.1%)	66 (24.7%)	365 (27.6%)		

Total percentages for some variables do not add up to 100 due to rounding. PHC, Primary Healthcare Center; CDC, Center for Disease Control and Prevention.

The status of physician wellbeing was evaluated across three dimensions. For physiological wellbeing, the mean self-rated health score (EQ-VAS) was 70.2 ± 16.7; regarding psychological wellbeing, 42.3% of physicians reported symptoms of anxiety or depression. No statistically significant differences were observed across the three institutional types for either of these dimensions (*p* = 0.061 and *p* = 0.465, respectively). Regarding work-related wellbeing, 72.4% of physicians did not meet the criteria for good work-related wellbeing under the primary strict definition, defined as simultaneously meeting all three criteria. Specifically, the rates for job satisfaction, work-life balance, and burnout were 53.2%, 39.4%, and 21.9%, respectively. Within the burnout sub-scales, the prevalence rates were 13.7% for emotional exhaustion, 13.6% for depersonalization, and 69.4% for low personal accomplishment. Significant differences were found in work-related wellbeing across institutions (*p* < 0.05). Physicians in primary healthcare facilities reported the highest rate of overall work-related wellbeing (33.9%). Hospital physicians had the highest rates of work-life imbalance (66.1%) and emotional exhaustion (15.6%), while those in CDCs reported the highest prevalence of low personal accomplishment (85.4%). Additional variables used in the expanded-adjustment sensitivity analyses, including facility category, professional field group, and years of practice, are summarized in Supplementary Table S1.

Table 2 presents the Spearman's correlation coefficients among the primary study variables. Weekly working hours were significantly inversely correlated with physiological wellbeing (*r* = −0.201, *p* < 0.001), psychological wellbeing (*r* = −0.143, *p* < 0.001), and work-related wellbeing under the primary strict definition (*r* = −0.233, *p* < 0.001). Weekly working hours were also positively correlated with monthly income (*r* = 0.207, *p* < 0.001). Regarding the relationship between monthly income and wellbeing, no statistically significant correlations were found with physiological, psychological, or work-related wellbeing in this bivariate analysis. Additionally, the three dimensions of multidimensional wellbeing were all positively inter-correlated, with coefficients ranging from 0.299 to 0.401 (*p* < 0.001).

**Table 2:** Spearman's correlations between working hours, monthly income, and multidimensional wellbeing.

Variables	1	2	3	4	5
1 Weekly working hours (10-h units)	1.000				
2 Monthly income	0.207^***^	1.000			
3 Physiological wellbeing	−0.201^***^	0.034	1.000		
4 Psychological wellbeing	−0.143^***^	0.01	0.401^***^	1.000	
5 Work-related wellbeing	−0.233^***^	−0.039	0.334^***^	0.299^***^	1.000

Weekly working hours is presented in 10-h units for consistency with the regression analyses. Correlation coefficients are unchanged by this linear rescaling. Work-related wellbeing refers to the primary strict definition.

* *p* < 0.05, ^**^p < 0.01, ^***^p < 0.001.

In multivariable regression analyses, weekly working hours showed consistent inverse associations with all three dimensions of wellbeing (Table 3). After adjustment for gender, age group, province, and institution type, each 10-h increase in weekly working hours was associated with lower physiological wellbeing (*B* = −5.057, *p* < 0.001), lower odds of psychological wellbeing (OR = 0.685, *p* < 0.001), and lower odds of work-related wellbeing under the primary strict definition (OR = 0.481, *p* < 0.001). These findings were consistent with H1. Consistent with H2a, weekly working hours were positively associated with monthly income (*B* = 0.049, *p* < 0.01; Table 3).

**Table 3:** Multivariable regression results for associations among weekly working hours, monthly income, and multidimensional wellbeing.

Variables	Mediator Model	Outcome Model A	Outcome Model B	Outcome Model C
	Monthly income (Log)	Physiological wellbeing	Psychological wellbeing	Work-related wellbeing
	***B (95% CI)***	***B (95% CI)***	***OR (95% CI)***	***OR (95% CI)***
Primary predictor				
Weekly working hours (per 10-h increase)	0.049^**^ (0.016, 0.083)	−5.057^***^ (−6.214, −3.899)	0.685^***^ (0.597, 0.785)	0.481^***^ (0.403, 0.574)
Mediator				
Monthly income (Log)	NA	3.630^***^ (1.734, 5.525)	1.487^**^ (1.168, 1.894)	1.259 (0.945, 1.675)
Control variables				
Gender (reference: Female)				
Male	0.079^**^ (0.024, 0.133)	2.365^*^ (0.533, 4.196)	0.839 (0.662, 1.064)	0.842 (0.641, 1.107)
Age (reference: < 35 years)				
35–49 years	0.159^***^ (0.102, 0.216)	−4.337^***^ (−6.213, −2.460)	0.639^**^ (0.496, 0.823)	0.875 (0.656, 1.167)
≥50 years	0.259^***^ (0.177, 0.340)	−1.656 (−4.693, 1.381)	0.713 (0.489, 1.039)	1.310 (0.875, 1.962)
Province (reference: Shaanxi)				
Henan	−0.223^***^ (−0.290, −0.156)	1.105 (−1.358, 3.568)	1.754^***^ (1.286, 2.392)	1.633^**^ (1.158, 2.305)
Shanghai	0.456^***^ (0.380, 0.532)	−3.935^**^ (−6.800, −1.069)	0.877 (0.614, 1.252)	0.805 (0.531, 1.220)
Tianjin	0.090^*^ (0.010, 0.170)	−0.769 (−3.464, 1.926)	1.146 (0.814, 1.613)	1.082 (0.725, 1.614)
Institution type (reference: PHCs)				
Hospitals	0.382^***^ (0.314, 0.451)	3.247^*^ (0.733, 5.761)	1.022 (0.737, 1.417)	1.269 (0.890, 1.810)
CDCs	0.228^***^ (0.160, 0.295)	1.038 (−1.637, 3.713)	0.697^*^ (0.490, 0.991)	0.576^**^ (0.389, 0.853)

Data are presented as unstandardized coefficients for the mediator model and model A (linear regression), and odds ratios (OR) for models B and C (logistic regression), with 95% confidence intervals (CI) in parentheses. The mediator model estimates the association between predictors and log monthly income.

PHC, Primary Healthcare Center; CDC, Center for Disease Control and Prevention. NA stands for “Not Applicable.”

* *p* < 0.05, ^**^p < 0.01, ^***^p < 0.001.

The GSEM-based mediation analysis with 5,000 bootstrap replications showed positive income-mediated indirect associations for physiological wellbeing (indirect association = 0.180; 95% bootstrap CI: 0.044, 0.368) and psychological wellbeing (indirect OR = 1.020; 95% bootstrap CI: 1.005, 1.041) (Table 4). These positive indirect associations were modest relative to the inverse direct associations between weekly working hours and wellbeing. For work-related wellbeing, the income-mediated indirect association was not statistically significant under the primary strict definition (indirect OR = 1.011; 95% bootstrap CI: 0.998, 1.032). Thus, the findings were consistent with H2b for physiological and psychological wellbeing, but not for work-related wellbeing under the primary strict definition.

**Table 4:** Analysis of overall, direct, and indirect associations through income between weekly working hours and multidimensional wellbeing.

Outcome	Association type	Estimate (95% *CI*)	**S.E**.
Model A Physiological wellbeing			
(Linear model)	Overall association	−4.877^***^(−6.031, −3.723)	0.588
	Direct association	−5.057 (−6.224, −3.862)	0.595
	Income-mediated indirect association	0.180 (0.044, 0.368)	0.080
Model B Psychological wellbeing			
(Logistic model)	Overall association	0.701^***^ (0.612, 0.803)	0.049
	Direct association	0.685 (0.594, 0.784)	0.049
	Income-mediated indirect association	1.020 (1.005, 1.041)	0.010
Model C Work-related wellbeing			
(Logistic model)	Overall association	0.489^***^ (0.410, 0.582)	0.044
	Direct association	0.481 (0.397, 0.571)	0.044
	Income-mediated indirect association	1.011 (0.998, 1.032)	0.009

Model A reports the overall, direct, and indirect associations for physiological wellbeing using a linear specification, whereas Models B and C report the corresponding associations for psychological and work-related wellbeing using logistic specifications. Work-related wellbeing refers to the primary strict definition. The first row in each model reports the estimate from the reduced-form regression without the mediator and represents the overall association between weekly working hours and the corresponding wellbeing outcome. The second and third rows report the model-based direct association and indirect association through income estimated using GSEM. The Estimate column displays unstandardized coefficients for Model A and odds ratios (ORs) for Models B and C. Weekly working hours were rescaled into 10-h units, and monthly income was log-transformed. All models adjusted for gender, age group, province, and institution type. The S.E. column reports robust standard errors for the reduced-form overall associations and bootstrap standard errors for the GSEM-based direct and income-mediated indirect associations. Confidence intervals for the reduced-form overall associations are based on robust standard errors, whereas confidence intervals for the direct and income-mediated indirect associations are percentile-based 95% bootstrap confidence intervals derived from 5,000 replications. For bootstrap-based direct and income-mediated indirect associations, statistical significance should be interpreted according to whether the percentile bootstrap confidence interval excludes the null value, which is 0 for linear coefficients and 1 for ORs. CI, confidence interval; GSEM, generalized structural equation modeling; OR, odds ratio.

* *p* < 0.05, ^**^p < 0.01, ^***^p < 0.001 for reduced-form overall associations.

Sensitivity analyses were generally consistent with the primary analyses (Supplementary Tables S2–S7). The main pattern of associations remained robust across the expanded-adjustment models, the cluster-robust sensitivity analysis, and the component-specific analyses of work-related wellbeing. In the cluster-robust analysis, the point estimate for the association between log-transformed monthly income and work-related wellbeing was unchanged, although the 95% confidence interval narrowly excluded 1 (OR = 1.259; 95% CI: 1.005, 1.576), indicating some sensitivity of statistical inference to the standard-error specification. In component-specific analyses, longer weekly working hours were associated with lower job satisfaction, poorer work-life balance, and higher risk of burnout, whereas higher income was associated with higher job satisfaction and lower risk of burnout, but showed no significant association with work-life balance. Using the relaxed definition of work-related wellbeing, the inverse association with weekly working hours remained evident (OR = 0.566, *p* < 0.001) (Supplementary Table S8). The corresponding mediation analysis under the relaxed definition is reported in Supplementary Table S9. Multicollinearity diagnostics are presented in Supplementary Table S10; VIFs for weekly working hours and log-transformed monthly income remained low across model specifications.

## Discussion

Physician wellbeing and healthcare service capacity are interdependent foundations of high-quality patient care and long-term healthcare system sustainability (1). Using data from 1,324 physicians working in hospitals, primary healthcare facilities, and CDCs across four province-level regions in China, this study examined the associations among working hours, income, and multidimensional wellbeing. Longer weekly working hours were consistently associated with poorer wellbeing across all three domains. The mediation analysis identified positive indirect statistical associations through monthly income for physiological and psychological wellbeing, whereas the corresponding indirect association for work-related wellbeing was not evident in the primary analysis but received some support in sensitivity analyses. These findings broadly align with the Job Demands-Resources theory, Conservation of Resources theory, and effort-reward perspectives. Situated within China's public healthcare compensation context, these findings provide a more nuanced understanding of how work demands and financial rewards are jointly associated with different domains of physician wellbeing, with implications for more targeted and sustainable healthcare workforce management.

Our study found that longer weekly working hours were consistently associated with poorer wellbeing across all three domains. This finding is in agreement with Tang et al. (42) and Jung et al. (43), who also reported poorer wellbeing among physicians working longer hours. However, Mendelsohn et al. (44) found no clear association between total weekly working hours and overall wellbeing or burnout. Differences in physician populations, working-time measures, and wellbeing outcomes may partly explain these variations. From the Job Demands-Resources and Conservation of Resources perspectives, extended working hours may restrict opportunities for recovery and gradually deplete physicians' physical and psychological resources. Physiologically, insufficient recovery time may contribute to cumulative fatigue and poorer perceived health. Psychologically, sustained cognitive and emotional demands may consume coping resources and contribute to symptoms of anxiety and depression (45). Regarding work-related wellbeing, long working hours may encroach on family, social, and leisure time, thereby intensifying emotional exhaustion and work-life conflict while limiting opportunities for resource replenishment (46).

We found that weekly working hours were positively associated with monthly income, in line with previous research (47). In Chinese public healthcare institutions, remuneration usually includes performance-related components linked to workload and service-related indicators (3). This compensation context may partly explain the observed positive association between longer weekly working hours and higher monthly income. The correspondence between greater work input and monetary reward is consistent with the effort-reward perspective.

Our mediation analysis identified positive indirect statistical associations through monthly income for physiological and psychological wellbeing, with some supportive evidence for work-related wellbeing in sensitivity analyses. Although preliminary bivariate analyses indicated no significant association between monthly income and any of the three wellbeing domains, higher monthly income was positively associated with physiological and psychological wellbeing after weekly working hours were incorporated into the structural model. This contrast may reflect a suppression effect, whereby the negative association between longer working hours and wellbeing masked the favorable association of higher income with these outcomes (48). Previous studies have similarly reported better job satisfaction and self-rated health among higher-earning physicians (33, 49), whereas lower income and financial hardship have been associated with greater burnout (50). Veenhoven's (51) absolute utility theory offers one explanation: higher income may improve access to healthcare, secure housing, and practical support such as household or childcare assistance, thereby reducing financial strain and preserving personal resources. These material and practical resources may help explain the positive indirect associations observed for physiological and psychological wellbeing.

In the primary model, the positive indirect association through monthly income was not evident for work-related wellbeing, although related sensitivity analyses provided some supportive evidence. The weaker and less consistent income-related association for work-related wellbeing may reflect the broader spectrum of professional experiences associated with burnout, job satisfaction, and work-life balance beyond monetary compensation alone. According to the effort-reward perspective (29), income constitutes merely one facet of occupational reward, operating alongside professional recognition, career prospects, job security, and perceived fairness. Additionally, prior studies indicate only modest correlations between pay and job satisfaction, as individuals evaluate financial returns in relation to their work effort, personal expectations, and peer comparisons (31, 33). Consequently, psychological adaptation and social comparison processes may attenuate the observed statistical correspondence between higher income and work-related wellbeing (52, 53).

The findings can also be viewed within the compensation context of Chinese public healthcare institutions. In settings where part of remuneration is tied to workload or service-related indicators, performance assessment may strengthen the perceived connection between work input and financial reward (8). Previous research suggests that strongly output-oriented or controlling assessment practices can be associated with lower professional autonomy and less intrinsic satisfaction derived from patient care (54, 55). These concerns may be particularly salient for physicians with strong public service motivation (PSM), for whom professional autonomy, altruistic service, and meaningful patient care are important sources of intrinsic work motivation (56). In this context, greater financial reward can coexist with concerns about professional autonomy and meaning at work, offering one possible explanation for the weaker income-related association observed for work-related wellbeing.

Theoretically, this study brings the Job Demands-Resources and effort-reward perspectives together within a single analytical framework. Weekly working hours represent a quantitative job demand, whereas monthly income represents a monetary reward associated with work input. The findings indicate that these two factors can be simultaneously associated with physician wellbeing in different directions. Moreover, the income-related indirect pathways varied across wellbeing domains, indicating that financial rewards may have different relevance for physiological, psychological, and work-related wellbeing. This domain-specific pattern supports examining physician wellbeing as a set of related but distinct outcomes.

Practically, the findings draw attention to several aspects of physician workforce management that warrant closer review. First, healthcare organizations and regulators could give greater attention to the systematic monitoring of consecutive duty hours, total weekly working time, and overtime not captured in formal schedules. Electronic attendance and scheduling systems could provide practical information for identifying repeated exceedance of predefined organizational thresholds and reviewing staffing levels, workload distribution, and shift arrangements. International standards such as the European Working Time Directive (EWTD) may offer useful reference points, with any local standards adapted to workforce capacity and service requirements (57). Second, the findings highlight the value of reviewing the balance between stable salary components and performance-related payments. Organizations and regulators could examine the transparency of payment criteria and whether performance evaluation gives appropriate consideration to service quality, patient experience, teamwork, and public-health responsibilities alongside activity-based indicators (58). Third, physician support strategies could give greater attention to non-financial aspects of the working environment, including occupational safety, career-development opportunities, professional recognition, and access to psychological and occupational-health services (59). These practices could be introduced through institution-specific pilot programs and evaluated before broader implementation.

From a broader social and public-health perspective, physician wellbeing is relevant to the stability of the healthcare workforce and the continuity of essential health services. Greater attention to sustainable working conditions and balanced remuneration arrangements may support physician retention, consistent service delivery, and healthcare-system resilience amid rising service demand and uneven workforce distribution.

There are several limitations in our study. First, the cross-sectional design calls for caution in interpreting temporal ordering and drawing causal inferences. The use of self-reported measures collected within a single survey may be subject to reporting and recall bias and may increase the potential for common-method bias. Working hours, income, and wellbeing were assessed over different reference periods, an issue that should also be considered when interpreting the assumed sequence from working hours to income to wellbeing. Second, the primary analyses focused on total monthly work-related income and did not evaluate specific compensation components or payment mechanisms. The findings therefore pertain to total monthly income. Third, residual confounding may remain because specialty-specific workload, management responsibilities, career stage, and other organizational conditions were not fully measured. Finally, purposive institution selection, quota sampling, and the oversampling of physicians from CDCs and primary healthcare centers limit the national representativeness of the sample.

Future research could use longitudinal or repeated-measures designs with aligned reference periods and combine survey responses with objective scheduling, payroll, and service-volume records. More detailed measures of compensation structures, perceived reward fairness, recovery opportunities, work-time control, and professional autonomy would allow the proposed mechanisms and domain-specific pathways to be examined more directly. Furthermore, probability-based sampling and population weighting would further strengthen representativeness and support comparisons across regions, specialties, and institution types.

## Conclusion

Among physicians surveyed in selected regions of China, longer weekly working hours were associated with poorer physiological, psychological, and work-related wellbeing. Monthly income played a modest statistical mediating role in the associations with physiological and psychological wellbeing, while the corresponding indirect association was not evident for work-related wellbeing under the primary strict definition. These findings highlight the relevance of considering work demands and income jointly when examining physician wellbeing. Further longitudinal and policy-evaluation studies are warranted to inform work-hour management and compensation reforms.

## Data Availability

The datasets presented in this article are not readily available because the datasets generated and analyzed during the current study are not publicly available due to ethical restrictions and the need to protect participant confidentiality. De-identified data may be made available from the corresponding author upon reasonable request and subject to appropriate ethics or institutional data governance approvals. Requests to access the datasets should be directed to xiaoyunliu@pku.edu.cn.
