# Supplementary material for: Extended working hours, income, and multidimensional wellbeing among physicians in China: a cross-sectional mediation analysis
Source: Front Public Health. 2026 Aug 26;14:1897935. doi: 10.3389/fpubh.2026.1897935 (PMC13553831; doi:10.3389/fpubh.2026.1897935)
Supplement: Supplementary file 1 [file Data_Sheet_1.pdf]

**Extended Working Hours, Income, and Multidimensional Wellbeing among Physicians in China: A Cross-sectional Mediation Analysis**

**supplementary tables**

**Supplementary Table S1. Descriptive Statistics of Facility Category, Broad Professional Field Group, and Years of Practice Used in the Expanded-Adjustment Sensitivity Analyses**

| Variables | N | % |
|---|---|---|
| **Facility category** | | |
| Primary healthcare centers | 301 | 22.7 |
| Secondary hospitals | 242 | 18.3 |
| Tertiary hospitals | 514 | 38.8 |
| Centers for Disease Control and Prevention | 267 | 20.2 |
| **Professional field group** | | |
| Public health | 276 | 20.8 |
| Primary care/general practice | 295 | 22.3 |
| Hospital medical specialties | 303 | 22.9 |
| Hospital surgical/procedural specialties | 328 | 24.8 |
| Other hospital specialties | 122 | 9.2 |
| **Years of practice**[a] | | |
| <10 years | 534 | 41.8 |
| 10–19 years | 439 | 34.4 |
| 20–29 years | 197 | 15.4 |

| | | |
|---|---|---|
| ≥30 years | 106 | 8.3 |

*Note: Percentages are presented as column percentages. [a]Percentages for years of practice were calculated among respondents with non-missing data (N = 1,276); 48 respondents had missing years-of-practice data. Facility category was used to incorporate hospital level by distinguishing primary healthcare centers, secondary hospitals, tertiary hospitals, and Centers for Disease Control and Prevention. Professional field group was constructed from institution type, license type, and department information. Years of practice was examined as an alternative indicator of career stage because it was conceptually related to age group. Abbreviations: CDC, Center for Disease Control and Prevention.*

**Supplementary Table S2. Expanded-Adjustment GSEM Estimates Using the Facility-Category Model**

| Variables | Mediator Model Monthly income (Log) B (95% CI) | Outcome Model A Physiological wellbeing B (95% CI) | Outcome Model B Psychological wellbeing OR (95% CI) | Outcome Model C Work-related wellbeing OR (95% CI) |
|---|---|---|---|---|
| **Primary predictor** | | | | |
| Weekly working hours (per 10-hour increase) | 0.038* (0.007, 0.069) | -5.098*** (-6.255, -3.941) | 0.674*** (0.587, 0.775) | 0.480*** (0.401, 0.573) |
| **Mediator** | | | | |
| Monthly income (Log) | NA | 3.681*** (1.668, 5.693) | 1.696*** (1.303, 2.207) | 1.305 (0.955, 1.782) |
| **Control variables** | | | | |
| Gender (reference: Female) | | | | |

| | | | | |
|---|---|---|---|---|
| Male | 0.058* (0.007, 0.109) | 2.371* (0.530, 4.213) | 0.832 (0.655, 1.057) | 0.858 (0.651, 1.129) |
| Age group (reference: <35 years) | | | | |
| 35–49 years | 0.006 (-0.059, 0.072) | -4.414*** (-6.724, -2.104) | 0.792 (0.572, 1.096) | 0.998 (0.698, 1.428) |
| ≥50 years | 0.089 (-0.007, 0.185) | -2.843 (-6.558, 0.873) | 0.821 (0.510, 1.323) | 1.584 (0.937, 2.677) |
| Province (reference: Shaanxi) | | | | |
| Henan | -0.194*** (-0.258, -0.131) | 1.483 (-0.985, 3.951) | 1.813*** (1.324, 2.483) | 1.683** (1.189, 2.381) |
| Shanghai | 0.412*** (0.338, 0.487) | -3.780* (-6.651, -0.909) | 0.899 (0.624, 1.295) | 0.761 (0.498, 1.163) |
| Tianjin | 0.043 (-0.034, 0.120) | -0.809 (-3.526, 1.908) | 1.217 (0.856, 1.730) | 1.039 (0.691, 1.562) |
| Facility category (reference: PHCs) | | | | |
| Secondary hospitals | 0.170*** (0.098, 0.242) | 3.521* (0.667, 6.376) | 1.076 (0.735, 1.576) | 1.361 (0.901, 2.057) |
| Tertiary hospitals | 0.295*** (0.212, 0.378) | 2.894 (-0.358, 6.146) | 1.258 (0.828, 1.912) | 0.985 (0.618, 1.569) |
| CDCs | 0.081* (0.012, 0.150) | 0.653 (-2.286, 3.591) | 0.823 (0.564, 1.200) | 0.526** (0.347, 0.796) |

| | | | | |
|---|---|---|---|---|
| Education (reference: Associate degree or below) | | | | |
| Bachelor's degree | 0.185** (0.054, 0.315) | -7.158** (-12.214, -2.103) | 0.393** (0.194, 0.792) | 0.729 (0.382, 1.391) |
| Postgraduate degree | 0.334*** (0.192, 0.476) | -5.892* (-11.346, -0.438) | 0.349** (0.165, 0.740) | 1.043 (0.513, 2.123) |
| Professional title (reference: Junior or below) | | | | |
| Intermediate | 0.212*** (0.147, 0.277) | -1.295 (-3.620, 1.030) | 0.677* (0.487, 0.941) | 0.824 (0.579, 1.174) |
| Senior | 0.394*** (0.306, 0.483) | 0.914 (-2.382, 4.210) | 0.607* (0.391, 0.944) | 0.785 (0.484, 1.273) |

*Note: Data are presented as unstandardized coefficients for the mediator model and Outcome Model A, and odds ratios (ORs) for Outcome Models B and C, with 95% confidence intervals (CIs) in parentheses. The mediator model estimates the association between predictors and log monthly income. The facility-category expanded model replaced institutional type with a facility-category variable distinguishing primary healthcare centers, secondary hospitals, tertiary hospitals, and Centers for Disease Control and Prevention, and additionally adjusted for education and professional title. Weekly working hours were rescaled into 10-hour units, and monthly income was log-transformed. Robust standard errors were used. Abbreviations: PHC, Primary Healthcare Center; CDC, Center for Disease Control and Prevention; CI, confidence interval; OR, odds ratio; NA, not applicable.* $^{*}p < 0.05$, $^{**}p < 0.01$, $^{***}p < 0.001$.

**Supplementary Table S3. Expanded-Adjustment GSEM Estimates Using the Broad Professional-Field Alternative Model**

| Variables | Mediator Model: Monthly income (Log), B (95% CI) | Outcome Model A: Physiological wellbeing, B (95% CI) | Outcome Model B: Psychological wellbeing, OR (95% CI) | Outcome Model C: Work-related wellbeing, OR (95% CI) |
|---|---|---|---|---|
| **Primary predictor** | | | | |
| Weekly working hours (per 10-hour increase) | 0.041* (0.009, 0.073) | -5.101*** (-6.272, -3.931) | 0.676*** (0.588, 0.777) | 0.470*** (0.393, 0.562) |
| **Mediator** | | | | |
| Monthly income (Log) | NA | 3.642*** (1.626, 5.659) | 1.708*** (1.314, 2.221) | 1.273 (0.936, 1.733) |
| **Control variables** | | | | |
| Gender (reference: Female) | | | | |
| Male | 0.062* (0.009, 0.115) | 2.202* (0.301, 4.103) | 0.819 (0.642, 1.046) | 0.818 (0.619, 1.081) |
| Age group(reference: <35 years) | | | | |
| 35–49 years | 0.003 (-0.063, 0.069) | -4.332*** (-6.652, -2.013) | 0.788 (0.569, 1.092) | 1.011 (0.709, 1.441) |
| ≥50 years | 0.091 (-0.006, 0.188) | -2.860 (-6.602, 0.882) | 0.818 (0.507, 1.318) | 1.550 (0.915, 2.625) |

| | | | | |
|---|---|---|---|---|
| Province (reference: Shaanxi) | | | | |
| Henan | -0.192*** (-0.256, -0.129) | 1.445 (-1.028, 3.919) | 1.806*** (1.320, 2.471) | 1.645** (1.163, 2.327) |
| Shanghai | 0.405*** (0.329, 0.481) | -3.637* (-6.531, -0.742) | 0.891 (0.620, 1.281) | 0.794 (0.522, 1.207) |
| Tianjin | 0.031 (-0.045, 0.106) | -0.853 (-3.588, 1.882) | 1.188 (0.836, 1.689) | 1.045 (0.696, 1.569) |
| Broad professional field group (reference: Public health) | | | | |
| Primary care/general practice | -0.066 (-0.134, 0.002) | -1.314 (-4.157, 1.529) | 1.231 (0.853, 1.777) | 1.704* (1.138, 2.550) |
| Hospital medical specialties | 0.148*** (0.078, 0.219) | 1.732 (-1.145, 4.610) | 1.307 (0.901, 1.896) | 1.995** (1.310, 3.036) |
| Hospital surgical/procedural specialties | 0.182*** (0.103, 0.261) | 2.621 (-0.316, 5.557) | 1.530* (1.037, 2.256) | 2.318*** (1.487, 3.614) |
| Other hospital specialties | 0.136* (0.028, 0.243) | 2.020 (-1.286, 5.326) | 1.480 (0.916, 2.391) | 2.152** (1.284, 3.606) |

| | | | | |
|---|---|---|---|---|
| Education (reference: Associate degree or below) | | | | |
| Bachelor's degree | 0.177** (0.046, 0.308) | -7.145** (-12.258, -2.032) | 0.393** (0.194, 0.795) | 0.742 (0.390, 1.413) |
| Postgraduate degree | 0.377*** (0.237, 0.518) | -6.260* (-11.628, -0.892) | 0.369** (0.175, 0.779) | 0.911 (0.453, 1.830) |
| Professional title (reference: Junior or below) | | | | |
| Intermediate | 0.209*** (0.143, 0.275) | -1.316 (-3.646, 1.014) | 0.671* (0.483, 0.932) | 0.825 (0.580, 1.173) |
| Senior | 0.398*** (0.309, 0.486) | 0.854 (-2.453, 4.161) | 0.608* (0.391, 0.946) | 0.780 (0.482, 1.261) |

*Note: Data are presented as unstandardized coefficients for the mediator model and Outcome Model A, and odds ratios (ORs) for Outcome Models B and C, with 95% confidence intervals (CIs) in parentheses. The mediator model estimates the association between predictors and log monthly income. The broad professional-field alternative model adjusted for gender, age group, province, broad professional field group, education, and professional title. Broad professional field group was constructed from institution type, license type, and department information and classified respondents into public health, primary care/general practice, hospital medical specialties, hospital surgical/procedural specialties, and other hospital specialties. Weekly working hours were rescaled into 10-hour units, and monthly income was log-transformed. Robust standard errors were used. Abbreviations: CI, confidence interval; OR, odds ratio; NA, not applicable. * $p < 0.05$, ** $p < 0.01$, *** $p < 0.001$.*

**Supplementary Table S4. Expanded-Adjustment GSEM Estimates Using the Years-of-Practice Alternative Model**

| Variables | Mediator Model: Monthly income (Log), B (95% CI) | Outcome Model A: Physiological wellbeing, B (95% CI) | Outcome Model B: Psychological wellbeing, OR (95% CI) | Outcome Model C: Work-related wellbeing, OR (95% CI) |
|---|---|---|---|---|
| **Primary Predictor** | | | | |
| Weekly working hours (per 10-hour increase) | 0.039* (0.007, 0.070) | -5.302*** (-6.472, -4.131) | 0.661*** (0.573, 0.762) | 0.466*** (0.387, 0.560) |
| **Mediator** | | | | |
| Monthly income (Log) | NA | 3.110** (1.138, 5.081) | 1.641*** (1.250, 2.155) | 1.346 (0.983, 1.844) |
| **Control Variables** | | | | |
| Gender (reference: Female) | | | | |
| Male | 0.069** (0.017, 0.120) | 2.393* (0.516, 4.271) | 0.877 (0.687, 1.119) | 0.859 (0.650, 1.135) |
| Years of practice (reference: <10 years) | | | | |
| 10–19 years | -0.018 (-0.083, 0.047) | -2.383 (-4.889, 0.123) | 0.707* (0.505, 0.990) | 1.032 (0.712, 1.494) |
| 20–29 years | 0.075 (-0.011, 0.161) | -2.516 (-5.967, 0.936) | 0.633* (0.403, 0.993) | 1.214 (0.732, 2.014) |

| | | | | |
|---|---|---|---|---|
| ≥30 years | 0.168** (0.061, 0.274) | -2.187 (-6.772, 2.399) | 0.749 (0.428, 1.312) | 1.823 (0.997, 3.334) |
| Province (reference: Shaanxi) | | | | |
| Henan | -0.204*** (-0.268, -0.139) | 0.860 (-1.590, 3.311) | 1.699** (1.229, 2.348) | 1.609** (1.124, 2.303) |
| Shanghai | 0.404*** (0.329, 0.478) | -4.261** (-7.177, -1.346) | 0.880 (0.607, 1.276) | 0.719 (0.467, 1.106) |
| Tianjin | 0.027 (-0.052, 0.106) | -1.642 (-4.409, 1.124) | 1.216 (0.847, 1.745) | 1.047 (0.691, 1.586) |
| Facility category (reference: PHCs) | | | | |
| Secondary hospitals | 0.178*** (0.105, 0.252) | 3.600* (0.702, 6.497) | 1.110 (0.751, 1.641) | 1.348 (0.884, 2.054) |
| Tertiary hospitals | 0.303*** (0.218, 0.388) | 2.488 (-0.807, 5.783) | 1.213 (0.790, 1.861) | 0.965 (0.600, 1.552) |
| CDCs | 0.076* (0.006, 0.145) | 0.759 (-2.197, 3.716) | 0.770 (0.523, 1.132) | 0.498** (0.328, 0.756) |
| Education (reference: Associate degree or below) | | | | |
| Bachelor's degree | 0.207** (0.065, 0.348) | -5.877* (-11.509, -0.246) | 0.372* (0.175, 0.794) | 0.843 (0.417, 1.707) |

| | | | | |
|---|---|---|---|---|
| Postgraduate degree | 0.374*** (0.218, 0.529) | -4.621 (-10.750, 1.507) | 0.306** (0.135, 0.691) | 1.228 (0.562, 2.685) |
| Professional title (reference: Junior or below) | | | | |
| Intermediate | 0.209*** (0.148, 0.270) | -2.103 (-4.437, 0.231) | 0.702* (0.509, 0.968) | 0.808 (0.568, 1.149) |
| Senior | 0.371*** (0.284, 0.458) | 0.369 (-2.981, 3.719) | 0.670 (0.426, 1.052) | 0.723 (0.437, 1.196) |

*Note: Data are presented as unstandardized coefficients for the mediator model and Outcome Model A, and odds ratios (ORs) for Outcome Models B and C, with 95% confidence intervals (CIs) in parentheses. The mediator model estimates the association between predictors and log monthly income. The years-of-practice alternative model replaced age group with years of practice group and adjusted for gender, province, facility category, education, and professional title. This model included 1,276 respondents because of missing data on years of practice. Weekly working hours were rescaled into 10-hour units, and monthly income was log-transformed. Robust standard errors were used. Abbreviations: PHC, Primary Healthcare Center; CDC, Center for Disease Control and Prevention; CI, confidence interval; OR, odds ratio; NA, not applicable. * p < 0.05, ** p < 0.01, *** p < 0.001.*

**Supplementary Table S5. Expanded-Adjustment Analysis of Direct and Indirect Associations Through Income Using the Facility-Category Model**

| Outcome | Association type | Estimate (95% CI) | S.E. |
|---|---|---|---|
| Model A: Physiological wellbeing | | | |
| | Overall association | -4.959*** (-6.123, -3.795) | 0.593 |
| | Direct association | -5.098 (-6.282, -3.947) | 0.593 |

| | | | |
|---|---|---|---|
| | Income-mediated indirect association | 0.139 (0.018, 0.307) | 0.074 |
| Model B: Psychological wellbeing | | | |
| | Overall association | 0.692*** (0.602, 0.794) | 0.049 |
| | Direct association | 0.674 (0.582, 0.772) | 0.048 |
| | Income-mediated indirect association | 1.020 (1.003, 1.044) | 0.011 |
| Model C: Work-related wellbeing | | | |
| | Overall association | 0.486*** (0.407, 0.580) | 0.044 |
| | Direct association | 0.480 (0.393, 0.569) | 0.044 |
| | Income-mediated indirect association | 1.010 (0.998, 1.029) | 0.008 |

*Note: Model A reports the overall, direct, and indirect associations for physiological wellbeing using a linear specification, whereas Models B and C report the corresponding associations for psychological and work-related wellbeing using logistic specifications. Work-related wellbeing refers to the primary strict definition. The first row in each model reports the estimate from the reduced-form regression without the mediator and represents the overall association between weekly working hours and the corresponding wellbeing outcome under the expanded adjustment model. The second and third rows report the direct association and income-mediated indirect association estimated using generalized structural equation modeling. The*

*Estimate column displays unstandardized coefficients for Model A and odds ratios (ORs) for Models B and C. The expanded adjustment model adjusted for gender, age group, province, facility category, education, and professional title. Facility category distinguished primary healthcare centers, secondary hospitals, tertiary hospitals, and Centers for Disease Control and Prevention. Weekly working hours were rescaled into 10-hour units, and monthly income was log-transformed. The S.E. column reports robust standard errors for the reduced-form estimates and bootstrap standard errors for the GSEM-based direct and indirect associations. Confidence intervals for the reduced-form estimates are based on robust standard errors, whereas confidence intervals for the direct and indirect associations are percentile-based 95% bootstrap confidence intervals derived from 5,000 replications. For bootstrap-based direct and indirect associations, statistical significance should be interpreted according to whether the percentile bootstrap confidence interval excludes the null value, which is 0 for linear coefficients and 1 for ORs. Abbreviations: CI, confidence interval; OR, odds ratio; PHC, Primary Healthcare Center; CDC, Center for Disease Control and Prevention. * $p < 0.05$, ** $p < 0.01$, *** $p < 0.001$ for reduced-form overall associations.*

**Supplementary Table S6. Sensitivity Analysis Using Cluster-Robust Standard Errors**

| Variables | Mediator Model Monthly income (Log) B (95% CI) | Outcome Model A Physiological wellbeing B (95% CI) | Outcome Model B Psychological wellbeing OR (95% CI) | Outcome Model C Work-related wellbeing OR (95% CI) |
|---|---|---|---|---|
| **Primary Predictor** | | | | |
| Weekly working hours (per 10-hour increase) | 0.049* (0.010, 0.089) | -5.057*** (-6.098, -4.015) | 0.685*** (0.608, 0.771) | 0.481*** (0.385, 0.601) |
| **Mediator** | | | | |

| | | | | |
|---|---|---|---|---|
| Monthly income (Log) | NA | 3.630** (1.569, 5.690) | 1.487*** (1.198, 1.847) | 1.259* (1.005, 1.576) |
| **Control Variables** | | | | |
| Gender (reference: Female) | | | | |
| Male | 0.079* (0.002, 0.155) | 2.365* (0.401, 4.328) | 0.839 (0.642, 1.098) | 0.842 (0.677, 1.049) |
| Age (reference: <35 years) | | | | |
| 35–49 years | 0.159*** (0.081, 0.237) | -4.337*** (-6.103, -2.571) | 0.639*** (0.507, 0.805) | 0.875 (0.725, 1.057) |
| ≥50 years | 0.259** (0.106, 0.412) | -1.656 (-4.330, 1.018) | 0.713 (0.486, 1.046) | 1.310 (0.814, 2.109) |
| Province (reference: Shaanxi) | | | | |
| Henan | -0.223** (-0.376, -0.069) | 1.105 (-0.955, 3.165) | 1.754** (1.170, 2.631) | 1.633* (1.084, 2.462) |
| Shanghai | 0.456*** (0.282, 0.629) | -3.935*** (-5.804, -2.065) | 0.877 (0.529, 1.452) | 0.805 (0.567, 1.144) |
| Tianjin | 0.090 (-0.034, 0.214) | -0.769 (-2.905, 1.368) | 1.146 (0.753, 1.744) | 1.082 (0.749, 1.563) |

| | | | | |
|---|---|---|---|---|
| Institution type (reference: PHCs) | | | | |
| Hospitals | 0.382*** (0.241, 0.524) | 3.247* (0.511, 5.983) | 1.022 (0.678, 1.541) | 1.269 (0.882, 1.825) |
| CDCs | 0.228** (0.078, 0.377) | 1.038 (-2.000, 4.075) | 0.697 (0.458, 1.061) | 0.576** (0.404, 0.822) |

*Note: Data are presented as unstandardized coefficients for the mediator model and Outcome Model A, and odds ratios (ORs) for Outcome Models B and C, with 95% confidence intervals (CIs) in parentheses. The mediator model estimates the association between predictors and log monthly income. Work-related wellbeing refers to the primary strict definition. Weekly working hours were rescaled into 10-hour units, and monthly income was log-transformed. Models adjusted for gender, age group, province, and institution type, with cluster-robust standard errors based on 25 clusters reflecting the organizational structure of participant recruitment. The mean cluster size was 53 respondents, with a range of 11 to 115.Abbreviations: PHC, Primary Healthcare Center; CDC, Center for Disease Control and Prevention; CI, confidence interval; OR, odds ratio; NA, not applicable. * p < 0.05, ** p < 0.01, *** p < 0.001.*

**Supplementary Table S7. Component-specific analyses of work-related wellbeing**

| Variables | Job satisfaction OR (95% CI) | Work-life balance OR (95% CI) | Absence of burnout OR (95% CI) |
|---|---|---|---|
| **Primary Predictor** | | | |
| Weekly working hours (per 10-hour increase) | 0.669*** (0.582, 0.770) | 0.493*** (0.422, 0.576) | 0.560*** (0.471, 0.665) |
| **Mediator** | | | |

| | | | |
|---|---|---|---|
| Monthly income (Log) | 1.962*** (1.531, 2.515) | 0.903 (0.700, 1.165) | 1.533** (1.171, 2.006) |
| **Control Variables** | | | |
| Gender (reference: Female) | | | |
| Male | 0.951 (0.751, 1.206) | 0.922 (0.719, 1.183) | 0.776 (0.586, 1.028) |
| Age (reference: <35 years) | | | |
| 35–49 years | 0.729* (0.566, 0.940) | 0.925 (0.711, 1.204) | 1.159 (0.859, 1.565) |
| ≥50 years | 1.088 (0.744, 1.593) | 1.458 (0.991, 2.144) | 1.642 (0.990, 2.723) |
| Province (reference: Shaanxi) | | | |
| Henan | 1.542** (1.132, 2.099) | 1.203 (0.875, 1.653) | 2.226*** (1.517, 3.267) |
| Shanghai | 0.667* (0.466, 0.954) | 1.076 (0.744, 1.557) | 0.618* (0.412, 0.926) |
| Tianjin | 1.036 (0.731, 1.469) | 1.151 (0.799, 1.659) | 1.051 (0.708, 1.560) |
| Institution type (reference: PHCs) | | | |

| | | | |
|---|---|---|---|
| Hospitals | 1.083 (0.777, 1.511) | 1.045 (0.756, 1.446) | 1.086 (0.719, 1.638) |
| CDCs | 0.468*** (0.328, 0.667) | 0.703* (0.496, 0.996) | 0.711 (0.459, 1.101) |

*Note: Values are presented as odds ratios (ORs) with 95% confidence intervals (CIs) in parentheses. Job satisfaction was coded as 1 for being satisfied or very satisfied with work. Work-life balance was coded as 1 for reporting agreement or strong agreement with having work-life balance. Absence of burnout was coded as 1 for reporting neither emotional exhaustion nor depersonalization burnout. Weekly working hours were rescaled into 10-hour units, and monthly income was log-transformed. Models adjusted for gender, age group, province, and institution type, consistent with the primary models. Robust standard errors were used. Abbreviations: PHC, Primary Healthcare Center; CDC, Center for Disease Control and Prevention; CI, confidence interval; OR, odds ratio. * p < 0.05, ** p < 0.01, *** p < 0.001.*

**Supplementary Table S8. Sensitivity analysis using a relaxed definition of work-related wellbeing: regression results**

| Variables | Mediator Model | Outcome Model D |
|---|---|---|
| | Monthly income (Log) | work-related wellbeing under the relaxed definition |
| | **B (95% CI)** | **OR (95% CI)** |
| **Primary Predictor** | | |
| Weekly working hours (per 10-hour increase) | 0.049**(0.016, 0.083) | 0.566***(0.491, 0.652) |
| **Mediator** | | |
| Monthly income (Log) | NA | 1.830***(1.426, 2.349) |
| Control Variables | | |
| Gender (Reference: Female) | | |
| Male | 0.079**(0.024, 0.133) | 0.917(0.720, 1.169) |
| Age (Reference:< 35 Years) | | |
| 35–49 years | 0.159***(0.102, 0.216) | 0.804(0.622, 1.040) |
| ≥50 years | 0.259***(0.177, 0.340) | 1.470(0.981, 2.204) |
| Province (Reference: Shaanxi) | | |
| Henan | -0.223***(-0.290, -0.156) | 1.462*(1.070, 1.998) |
| Shanghai | 0.456***(0.380, 0.532) | 0.752(0.523, 1.079) |
| Tianjin | 0.090*(0.010, 0.170) | 0.966(0.679, 1.375) |

| | | |
|---|---|---|
| Institution type (Reference: PHCs) | | |
| Hospitals | 0.382***(0.314, 0.451) | 1.067(0.761, 1.494) |
| CDCs | 0.228***(0.160, 0.295) | 0.573**(0.401, 0.820) |

*Note: This table presents a sensitivity analysis using a relaxed definition of work-related wellbeing. The relaxed definition classified respondents as having work-related wellbeing if they met at least two of the three favorable component criteria: job satisfaction, work-life balance, and absence of emotional exhaustion or depersonalization burnout. This differs from the primary strict definition, which required all three favorable criteria to be met simultaneously. Weekly working hours were rescaled into 10-hour units, and monthly income was log-transformed. Models adjusted for gender, age group, province, and institution type. Robust standard errors were used. Abbreviations: PHC, Primary Healthcare Center; CDC, Center for Disease Control and Prevention; CI, confidence interval; OR, odds ratio; NA, not applicable. * $p < 0.05$, ** $p < 0.01$, *** $p < 0.001$.*

**Supplementary Table S9. Sensitivity analysis using a relaxed definition of work-related wellbeing: mediation analysis**

| Outcome | Association Type | Estimate (95% CI) | S.E. |
|---|---|---|---|
| Model D: work-related wellbeing under the relaxed definition | | | |
| (Logistic Model) | Overall association | 0.590***(0.514, 0.678) | 0.042 |
| | Direct association | 0.566(0.489, 0.648) | 0.041 |
| | Income-mediated indirect association | 1.030(1.009, 1.058) | 0.013 |

*Note: This table presents a sensitivity analysis of overall, direct, and indirect associations through income using the relaxed definition of work-related wellbeing. The relaxed definition classified respondents as having work-related wellbeing if they met at least two of the three favorable component criteria: job satisfaction, work-life balance, and absence of emotional exhaustion or depersonalization burnout. This differs from the primary strict definition, which required all three favorable criteria to be met simultaneously. The first row reports the estimate from the reduced-form regression without the mediator and represents the overall association between weekly working hours and work-related wellbeing under the relaxed definition. The second and third rows report the direct association and income-mediated indirect association estimated using generalized structural equation modeling. The Estimate*

*column displays odds ratios (ORs). Weekly working hours were rescaled into 10-hour units, and monthly income was log-transformed. The model adjusted for gender, age group, province, and institution type. The S.E. column reports robust standard errors for the reduced-form estimate and bootstrap standard errors for the GSEM-based direct and indirect associations. Confidence intervals for the reduced-form estimate are based on robust standard errors, whereas confidence intervals for the direct and indirect associations are percentile-based 95% bootstrap confidence intervals derived from 5,000 replications. Statistical significance for bootstrap-based direct and indirect associations should be interpreted according to whether the percentile bootstrap confidence interval excludes 1. Abbreviations: CI, confidence interval; OR, odds ratio. * $p < 0.05$, ** $p < 0.01$, *** $p < 0.001$ for the reduced-form overall association.*

**Supplementary Table S10. Variance Inflation Factor Diagnostics for the Primary and Expanded-Adjustment Models**

| Adjustment model | N | Weekly working hours VIF | Log monthly income VIF | Mean VIF | Maximum VIF |
|---|---|---|---|---|---|
| Primary model | 1,324 | 1.43 | 1.45 | 1.53 | 2.13 |
| Facility-category expanded model | 1,324 | 1.45 | 1.65 | 2.82 | 9.35 |
| Professional-field alternative model | 1,324 | 1.45 | 1.64 | 2.65 | 8.97 |
| Years-of-practice alternative model | 1,276 | 1.44 | 1.69 | 2.82 | 9.98 |

*Note: VIFs were estimated for each adjustment set using the physiological wellbeing model. Because VIFs depend on the predictor matrix and analytic sample, the diagnostics also apply to corresponding outcome models using the same predictors and sample. For categorical predictors, Stata reports category-specific VIFs for the indicator variables. Elevated VIFs in the expanded models were concentrated in education indicators. VIFs for weekly working hours, log-transformed monthly income, and all other predictors remained below 3.50. Abbreviation: VIF, variance inflation factor.*
